# Mitochondrial Genome Sequencing in Mesolithic North East Europe Unearths a New Sub-Clade within the Broadly Distributed Human Haplogroup C1

**DOI:** 10.1371/journal.pone.0087612

**Published:** 2014-02-04

**Authors:** Clio Der Sarkissian, Paul Brotherton, Oleg Balanovsky, Jennifer E. L. Templeton, Bastien Llamas, Julien Soubrier, Vyacheslav Moiseyev, Valery Khartanovich, Alan Cooper, Wolfgang Haak, The Genographic Consortium

**Affiliations:** 1 Australian Centre for Ancient DNA, School of Earth and Environmental Sciences, University of Adelaide, Adelaide, South Australia, Australia; 2 Vavilov Institute for General Genetics, Russian Academy of Sciences, Moscow, Russia; 3 Research Centre for Medical Genetics, Russian Academy of Medical Sciences, Moscow, Russia; 4 Peter the Great Museum of Anthropology and Ethnography (Kunstkamera) RAS, St Petersburg, Russia; University of Perugia, Italy

## Abstract

The human mitochondrial haplogroup C1 has a broad global distribution but is extremely rare in Europe today. Recent ancient DNA evidence has demonstrated its presence in European Mesolithic individuals. Three individuals from the 7,500 year old Mesolithic site of Yuzhnyy Oleni Ostrov, Western Russia, could be assigned to haplogroup C1 based on mitochondrial hypervariable region I sequences. However, hypervariable region I data alone could not provide enough resolution to establish the phylogenetic relationship of these Mesolithic haplotypes with haplogroup C1 mitochondrial DNA sequences found today in populations of Europe, Asia and the Americas. In order to obtain high-resolution data and shed light on the origin of this European Mesolithic C1 haplotype, we target-enriched and sequenced the complete mitochondrial genome of one Yuzhnyy Oleni Ostrov C1 individual. The updated phylogeny of C1 haplogroups indicated that the Yuzhnyy Oleni Ostrov haplotype represents a new distinct clade, provisionally coined “C1f”. We show that all three C1 carriers of Yuzhnyy Oleni Ostrov belong to this clade. No haplotype closely related to the C1f sequence could be found in the large current database of ancient and present-day mitochondrial genomes. Hence, we have discovered past human mitochondrial diversity that has not been observed in modern-day populations so far. The lack of positive matches in modern populations may be explained by under-sampling of rare modern C1 carriers or by demographic processes, population extinction or replacement, that may have impacted on populations of Northeast Europe since prehistoric times.

## Introduction

Human mitochondrial haplogroup (hg) C is part of the non-African macro-haplogroup M. Most of the diversity of hg C is found today in indigenous populations of Asia and the Americas [1]–[2]. In northern Asia, hg C represents, together with hg D, more than half of the present-day mitochondrial (mtDNA) diversity [3]. Haplogroup Z, the sister-clade of hg C, has a broad distribution ranging from northern Scandinavia (in Saami) to central Asia, Siberia, northern China and Korea.

Phylogenetic analyses of complete mtDNA genomes revealed four major sub-clades of hg C, termed C1, C4, C5 and C7 (e.g., [3]–[7]). Of these, haplogroup C1 has one of the broadest distributions of all human mtDNA hgs in the world, ranging from Iceland to East Asia and the Americas. The C1 basal haplotype is defined by the hypervariable region I and II (HVR-I and HVR-II) motif: A16129G, T16187C, C16189T, G16230A, T16278C, T16298C, C16311T, T16325C, C16327T (HVR-I; numbering according to the Reconstructed Sapiens Reference Sequence RSRS; [8]) and C146T, C152T, C195T, A247G, A249d, 290–291d and T489C (HVR-II).

**Figure 1:**
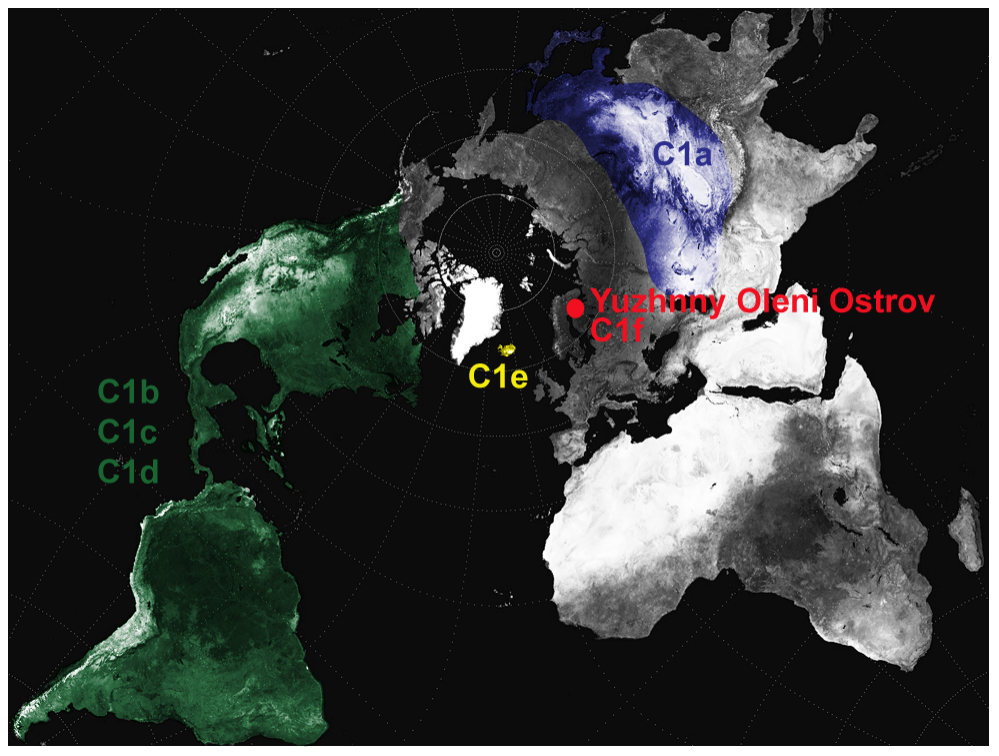
Approximate geographical distribution of the C1 sub-clades in modern and Mesolithic Yuzhnyy Oleni Ostrov populations.

The phylogeny of hg C1 is structured into five distinct monophyletic sub-clades, C1a, C1b, C1c, C1d and C1e, which exhibit a clear geographical distribution pattern ([4], [7], [9]–[10]; Figure 1). Three of the C1 sub-clades (C1b, C1c and C1d) are restricted to Native American populations, although spread widely across the American continent [11]–[12]. It was proposed that these three Native American C1 sub-clades were among the ancestral founder lineages, along with hg A2, B2 and D1, which reached the Americas during the initial human colonisation of the continent [4]–[5], [7], [9]. The source population of this migration was assumed to be in eastern Asia, where most of the diversity of hg C is observed today, and where C1a, a sister clade of the American C1 clades, is found at low frequencies in diverse indigenous populations [9]. The peopling of the Americas was made possible by the Beringian ice-free land bridge that connected north-east Siberia and Alaska before (∼30,000 years Before Present, BP) and after (∼13,000 yBP) the last Ice Age [9], [13]. The place of origin of ancestral hg C1 was approximated in the Amur River region just south of Beringia (eastern Asia) on the basis of the current frequency distribution of hg C1 in Asia [9]. The last hg C1 clade to have been described, C1e, was only found recently in a few individuals in Iceland and was shown to be distinct from any of the previously defined Asian and American clades on the basis of seven coding region and three control region mutations [10].

**Figure 2:**
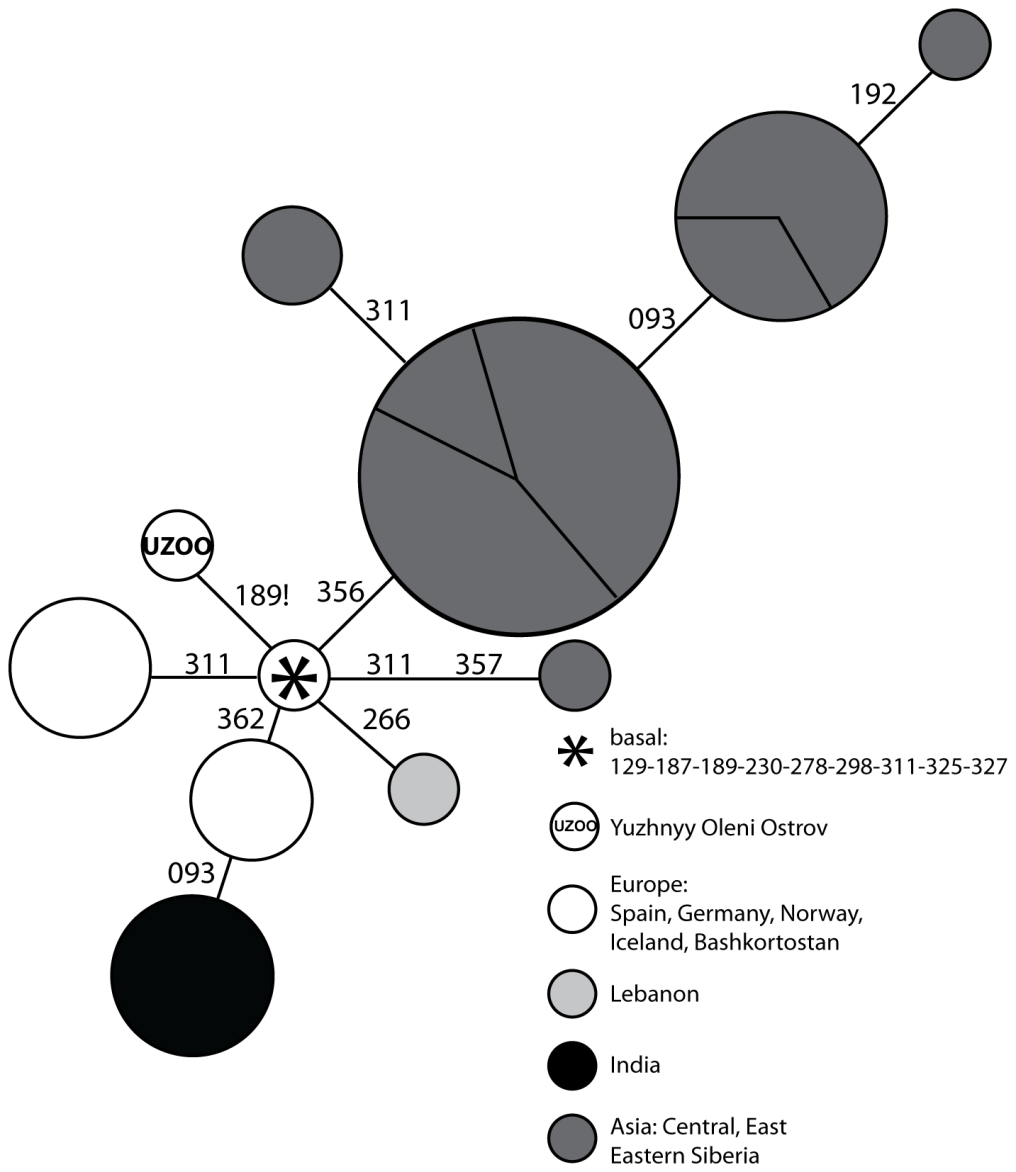
Network representation of C1 HVR-I sequences in Mesolithic Yuzhnyy Oleni Ostrov and modern Eurasian populations. Each haplotype is represented by a circle, the area of which is proportional to the number of individuals that were found to carry this haplotype in the literature. The haplotypes are colour-coded according to their geographical location: India (black), Asia (dark grey), Lebanon (light grey), and Europe (white). Each section of the circles represents individuals sampled from a same population. Mutations are all substitutions and are reported according to the Reconstructed Sapiens Reference Sequence minus 16000. The star represents the hypervariable region-I haplotype that characterizes the root of the C1 clade. The haplotype labeled ‘UZOO’ is the hypervariable region-I haplotype sequenced from individuals of the archaeological site of Yuzhnyy Oleni Ostrov. All the other haplotypes were found in modern populations.

In Europe, the dense and extensive sampling of the HVR-I diversity has revealed extremely low frequencies of hg C1, with very few haplotypes found in Germans [14], Canarians [15], Icelanders [16]–[17] and Bashkirs [18] (Figure 2). These sequences lack HVR-I Single Nucleotide Polymorphisms (SNPs) diagnostic of the sub-clades C1a (T16356C) and C1d (A16051G). However, a more detailed assignment of the European haplotypes into sub-haplogroups is limited by the low resolution provided by HVR-I and the lack of information from the coding region thus far. These limitations therefore impede the reconstruction of their precise phylogenetic placement, origin and relation to the Asian, American and Icelandic sister-clades.

Three hypotheses for the origins of the C1 lineages in Europe can be put forward [10], [16]–[17]. Hypothesis 1 proposes a recent genetic input from Asia into Europe during historical times. Historically, Central and East Europe experienced repeated influences from invading groups from the neighbouring Asian steppes, which could have introduced C1 into Europe. Well-documented examples include the Huns from Mongolia in the 4^th^–5^th^ centuries Anno Domini (A.D.) and the Mongols in the 13^th^ century A.D. [19]. However, the common Asian C1a clade is characterised by the HVR-I transition T16356C, which has not been found in any European C1 haplotype. In the case of a recent Asian ancestry, a reversal of the mutation at nucleotide position (np) 16356 in all the European sequences would be required. Hypothesis 2 assumes an American origin, where hg C1 would have reached Europe through admixture between Native Americans and Europeans. This gene flow may have occurred during and after the colonization of the New World by Europeans in the 15^th^ century A.D., i.e. in post-Columbian times [16]–[17]. Alternatively, one explanation for the presence of the sub-clade C1e in Iceland was a pre-Columbian admixture between Native Americans and Icelandic Vikings, which are widely acknowledged to have built temporary pioneer settlements in the north-western coast of the Americas in the 10^th^ century A.D. [10]. In accordance with the hypothesis of the American origin, few European C1 HVR-I sequences could belong to either the C1b or C1c American clades, as diagnostic SNPs for these two clades are located outside HVR-I [16]–[17]. Hypothesis 3, proposes that hg C1 has been present in Europe since prehistoric times in the light of the recent finding of hg C1 HVR-I haplotypes in three individuals of the 7,500-year-old Mesolithic site of Yuzhnyy Oleni Ostrov (individuals UZOO-7, UZOO-8, and UZOO-74), North West Russia (Figure 1; [20]). The classification of the corresponding mtDNA haplotype within hg C1 was previously determined by HVR-I sequencing (hg C1 defining mutation T16325C) as well as by typing informative SNPs in the coding region (hg C defining mutation A13263G [20]), but the lack of resolution of the HVR-I sequence prevented establishing clear phylogenetic relationships with currently known hg C1 clades.

In this study, we sequenced the complete mtDNA genome of one of the three Mesolithic hg C1 carriers from the Yuzhnyy Oleni Ostrov archaeological site (individual UZOO-74) in order to shed further light on the population history of the Yuzhnyy Oleni Ostrov hunter-gatherers and to contribute to the characterisation of the mtDNA diversity, evolutionary history and phylogeography within hg C1.

To this day, complete mtDNA genome sequences from ancient specimens have successfully been determined for a Palaeo-Eskimo from Greenland [21], the 5,000-year-old Tyrolean Iceman [22], a 700-year-old individual from New Zealand [23], a Palaeolithic individual from Tianyan, China [24], as well as several Palaeolithic, Mesolithic and Neolithic individuals from Europe [25]–[28]. Most of these mtDNA genomes were obtained on high-throughput ‘next-generation’ sequencing platforms (e.g., [29]). In accordance with these recent studies, we first created a genomic library, which was subsequently enriched for mtDNA in two iterative rounds of hybridisation to in-house designed biotinylated DNA probes, following the protocol by [28]. The enriched DNA library was sequenced on an Ion Torrent PGM platform. We analysed the resulting mtDNA genome from the Yuzhnyy Oleni Ostrov specimen in the light of an updated phylogeny of all currently available hg C1 lineages. The resulting mtDNA genome sequence allowed us to identify a novel C1 sub-clade, coined “C1f”, which fills a gap in the knowledge of the hg C1 distribution in West Eurasia.

## Results

### Ancient Mitochondrial Genome Sequencing

Our ancient DNA (aDNA) enrichment, followed by sequencing on an Ion Torrent PGM platform, allowed the unambiguous determination of 99.8% (16537 out of 16569 base pairs, bp) of the UZOO-74 mtDNA genome with 20,579 unique reads assembled to the RSRS at an average coverage of 68X (Table 1) and average read length of 55±14.5 bp. Indels, a well-defined homopolymer sequencing error, were observed in the resulting data set. However, adequate depth and coverage of the mtDNA genome sequence data prevented false-positive base calls.

**Table 1:** Positions and nucleotide changes in the Yuzhnyy Oleni Ostrov C1f haplotype when compared to the Reconstructed Sapiens Reference Sequence.

**Coverage**	99.8%
**Missing bases**	np 7525–7556
**Average redundancy**	68.5 (23.2 stdv)
**Minimum redundancy**	0
**Maximum redundancy**	126
**Mutations**	C146T, C152T, T182C!, C195T, **G247A!**, A249d, 290–291d, T489C, *522.AC*, A769G, A825t, A1018G, A2758G, C2885T, T3552a, T3594C, G4104A, T4312C, A4715G, G7146A, C7196a, T7256C, A7521G, T8468C, **A8577G**, G8584A, T8655C, A9545G, C10400T, T10664C, A10688G, C10810T, C10915T, **A11605t**, G11914A!, **A12217G**, G13105A, A13263G, G13276A, T13506C, T13650C, T14318C, T14783C, G15043A, G15301A, A15487t, A16129G, *A16183c*, T16187C, **T16189C!,** G16230A, T16278C, T16298C, C16311T, T16325C, C16327T, *C16519T*

Nucleotide changes in bold represent mutations in the Yuzhnyy Oleni Ostrov haplotype that are new within the C1 clade. Transitions are reported with upper case letters and transversions with lower case letters. “!” indicates a back mutation. np, nucleotide position. stdv, standard deviation.

Missing data comprised 32 consecutive bp, spanning nps 7525–7556 (Table 1). Other mtDNA genome sequences that we have generated following the same protocol have also exhibited a low coverage or dropout in exactly the same region [28]. Figure S1 shows that low GC content regions are characterised by a poor coverage; in particular in the region 7525–7556, GC content is only 25.0% compared to 44.4% GC for the whole mtDNA. It is therefore suspected that this region of the mtDNA genome is energetically sub-optimal (AT-rich) for two rounds of hybridisation and stringency washes in the ionic and temperature conditions used here [28], which may produce secondary DNA structures that adversely affect hybridisation-based DNA capture [30]. In addition, re-amplification of the enriched libraries was done with AmpliTaq Gold (Applied Biosystems; see [28]), a *Taq* polymerase known to be biased towards high GC content [31].

The ancient mtDNA haplotype of individual UZOO-74 differed from the RSRS at 58 nucleotide positions (Table 1). Among these, 51 substitutions define the sub-hg C1 in accordance with the current phylogeny (www.PhyloTree.org), including back mutations at T182C! and G11914A!, which are identical by state to the RSRS. In addition, UZOO-74 showed five private substitutions (G247A!, A8577G, A11605t, A12217G and T16189C!, including two additional back mutations in the hypervariable region). These five additional nucleotide differences were directly amplified and sequenced from two different extracts in order to verify whether they represented true private mutations defining a novel C1 sub-clade (Table S1). All five mutations have been confirmed by direct sequencing and were taken into account in further phylogenetic analysis of hg C1 sequences. Importantly, we did not observe any SNPs characteristic of other hgs, nor any mixed signals that could indicate systematic DNA degradation or DNA contamination from exogenous sources.

We analysed the pattern of nucleotide misincorporation at the 3′ and 5′-ends of the DNA fragments in order to assess whether the estimated age of the molecules reflects the age of the sample [32]–[33]. We observed a C-to-T substitution frequency of 22.4% at the 5′-end (Figure S2), which sits well with previous findings that suggested a correlation between this frequency and the age of the samples [34], whereby samples older than 500 years had a C-to-T substitution frequency >10%. The Bayesian statistical framework implemented in mapDamage v2.0.1 [33] also provided simulated posterior distribution of three parameters of the damage model: λ, probability of terminating in overhang; ∂D, probability of cytosine deamination in double strands; and ∂S, probability of cytosine deamination in single strands. The posterior distribution of these parameters all departed from 0 (λ: mean, 0.582; standard deviation, 0.021; ∂D: mean, 0.036; standard deviation, 0.001; ∂S: mean, 0.651; standard deviation, 0.041), in accordance with the DNA sequences generated from the UZOO-74 individual arising from aDNA molecules, and not from contamination by more recent DNA molecules during post-excavation handling. The results of the DNA damage analyses support the authenticity of the aDNA data presented here.

### The Sequence from Mesolithic Yuzhnyy Oleni Ostrov Defines a Novel Lineage within the C1 Phylogeny

Upon confirmation of the five novel mutations in the C1 mtDNA genome of individual UZOO-74, we genotyped the same SNPs in the two other C1 individuals UZOO-7 and UZOO-8 from Yuzhnyy Oleni Ostrov. Direct sequencing confirmed the presence of all five novel SNPs, suggesting that the three C1 individuals from Yuzhnyy Oleni Ostrov were maternally related. Their mtDNA genomes may be strictly identical, or they may display differences in the form of additional private SNPs at coding region positions that have not been sequenced in these remaining individuals. The precise nature of the genetic relationships between these individuals cannot be inferred from the archaeological and genetic data currently available.

**Figure 3:**
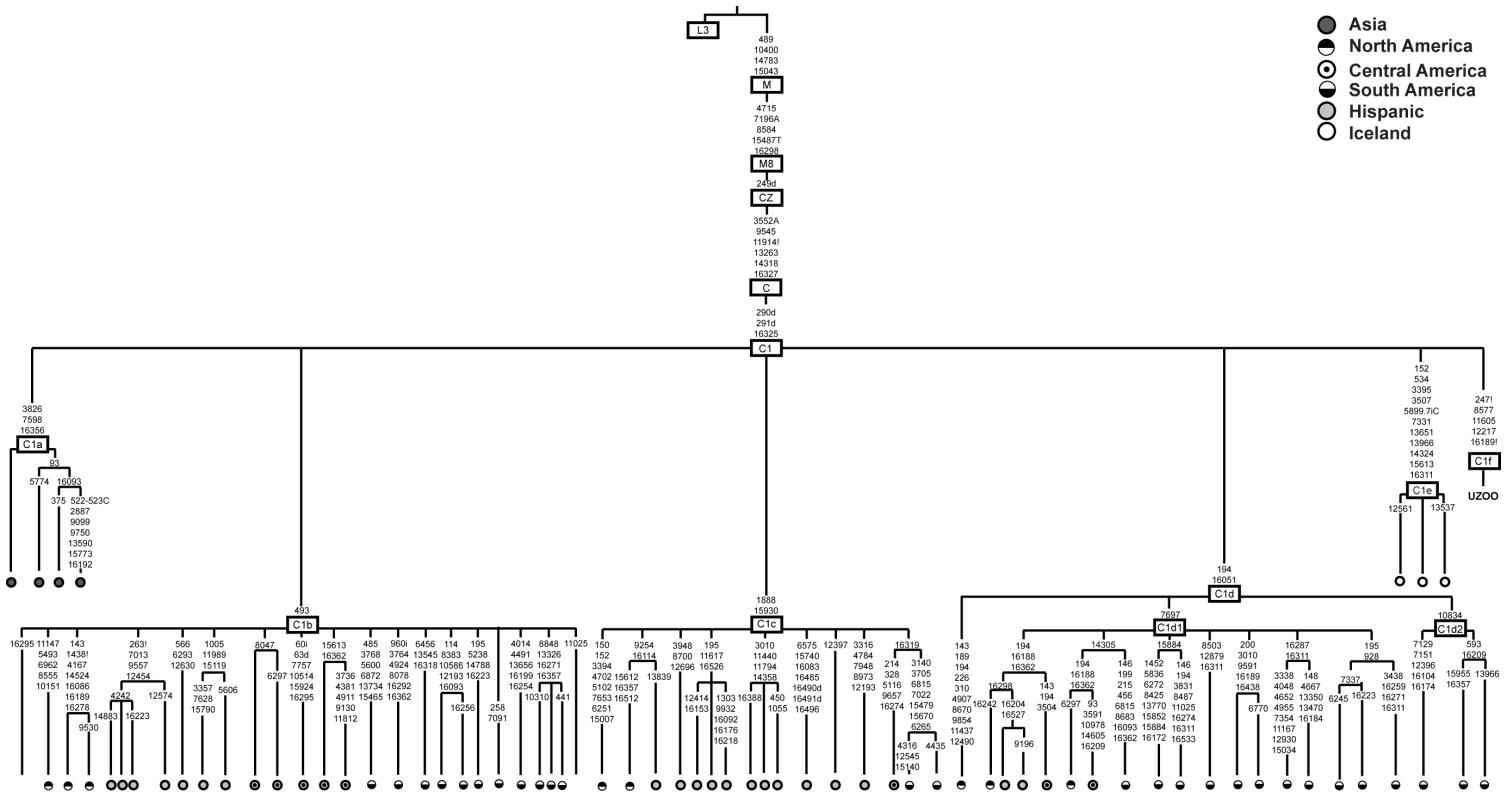
Median joining phylogenetic tree of haplogroup C1 complete mitochondrial genomes. A haplogroup sequence L3 sequence was chosen as the root of the tree. Mutations are reported according to the Reconstructed Sapiens Reference Sequence. “d” represents deletions. “i” represents insertions.

A search against the public Phylotree database yielded no match for the newly sequenced C1 mtDNA haplotype in 16810 modern complete mtDNA genomes (entries, mtDNA tree build 15 (30 Sep 2012) on PhyloTree.org [35]). The five SNPs identified in these individuals, and among these, the three coding region mutations A8577G, A11605t and A12217G in particular, represent novel sub-clade defining mutations that have not been reported together within a single hg C1 haplotype before. We therefore assigned them to a distinct new clade, which we tentatively named “C1f” following the conventional nomenclature (Figure 3). The resulting phylogenetic reconstruction shows that clade C1 is now characterised by six monophyletic sub-clades: C1a, C1b, C1c, C1d, C1e and C1f. The tree topology suggests that the Eurasian C1 sub-clades, the East Asian C1a, the rare C1f branch from Yuzhnyy Oleni Ostrov and the Icelandic C1e split early from the most recent common ancestor of the C1 clades and evolved independently (Figure 3).

## Discussion

### Under-sampling of the Mitochondrial Genome Diversity

In the present study, we established that the hg C1 mtDNA genome sequence carried by the Mesolithic individuals of the Yuzhnyy Oleni Ostrov site in north-western Russia defines a new clade, C1f, within the hg C1 phylogeny. Because of the polytomous topology of the hg C1 tree, no direct phylogenetic relationship could be established between C1f and the other well geographically defined C1 clades. As a result, clear inferences regarding the origin and evolutionary history of the C1f clade will remain difficult to draw, unless future sequencing of complete mtDNA genomes uncovers sequences closely related to the C1f genome sequenced here.

The absence of a direct match with sequences in databases of complete mtDNA genomes could be explained by under-sampling of mtDNA genomes in modern human populations. The number of published modern-day *Homo sapiens* complete mtDNA genome sequences is still small compared to that of HVR-I sequences. As such it is not too surprising that studies regularly report the discovery of novel clades and lineages (e.g., hg C1e [10]; within hg C1d [7]). Furthermore, the geographical coverage of modern-day populations for complete mtDNA genome sequencing is still unequally distributed, and the sampling so far has focused either on few specific populations or on particular hgs (e.g., [36]–[38]). As a consequence, mtDNA genomes available from the literature can still only provide an incomplete yet biased picture of the full, extant mtDNA diversity.

### Absence of Match for C1f in Asia

Asia, and more precisely Siberia, could be considered as potential places of origin for the C1f clade identified in the Mesolithic site of Yuzhnyy Oleni Ostrov. This hunter-gatherer group was indeed shown to exhibit mtDNA affinities with modern-day populations of western and southern Siberia, the Altai region, or Mongolia [20]. The hypothesis of an Asian origin for the C1f sub-clade is also supported by the fact that most of the diversity of hg C is found in present-day populations of East Eurasia [3]. Sequences closely related to hg C1f may persist in modern-day populations of East Eurasia but remain undetected to date, as mtDNA genomes for these populations have not been as densely and extensively sampled as, for example, European populations.

### Absence of Match for C1f in Europe

Despite the dense sampling of mtDNA in modern-day populations of Europe, only a few hg C1 HVR-I and no hg C1f mtDNA genome sequences were detected. The close matches for the HVR-I sequence of C1f did not display the back mutation T16189C! (Figure 2) and hence, none matched the C1f HVR-I haplotype exactly. However, np 16189 has been described as one of the top five transitional hotspots in the human control region [39], and hence provides little phylogenetic discrimination power. It is possible that these European haplotypes belong to the C1f clade without harbouring the mutation at np 16189. Therefore, additional SNPs in the coding region are required to definitely rule out these Eurasian C1 haplotypes as potential members of the C1f clade, and potential persistence of hg C1f in Europe since the Mesolithic.

### Extinction or Near-extinction of C1f due to Post-Mesolithic Population Dynamics

Low frequencies and a restricted distribution seem to have been characteristic of hg C1 already in Mesolithic times, as hg C1 could not be detected in any of the other European Mesolithic populations sampled for ancient mtDNA in Eurasia further west [20]: in central/eastern Europe [25], [27], [40], and in Scandinavia [41]. This suggests an under-sampling of Mesolithic populations for aDNA, mating isolation of the Yuzhnyy Oleni Ostrov population, and/or influences from Siberian populations that had not reached Central Europe. Because of its low frequency, the distribution of hg C1 is prone to be affected by demographic processes, such as genetic drift or population replacements that may have occurred since Mesolithic times. Eventually, hg C1 may have reached extremely low frequencies or have gone extinct, thus preventing it from being detected in present-day European populations. The effects of these population processes can be observed at the population level, as the Yuzhnyy Oleni Ostrov group, similarly to the other described Mesolithic populations of Europe, was indeed shown to exhibit little genetic continuity with present-day Europeans [20]. Significant dissimilarities have been shown between the mtDNA gene pool of European Mesolithic populations characterised by a low diversity and high frequencies of hg U sub-clades (U2, U4, U5 and U8 in particular), and the rather homogeneous mtDNA makeup of present-day Europeans, which arrived during the Neolithic transition and subsequent periods [20], [40]–[42].

### Absence of Match for C1f in the Americas

The Americas also remain under-sampled for complete mtDNA genomes and could be suggested as a potential geographical origin for the C1f lineage, as it has been for the Iceland-restricted C1e sub-clade [10]. For C1e, an American origin through mating of Viking explorers with Native American women sometime earlier than 300 years ago was proposed by [10]. Among other hypotheses including that of a European origin, an American origin was favoured on the basis that most of the hg C1 diversity is found on the American continent, despite the fact that no sequence belonging to hg C1e could be detected in the Americas (or anywhere else). This lack of match was explained by under-sampling of the American mtDNA genome diversity [10]. In any case, if admixture between Native Americans and Vikings did occur, it must have been limited, as no other American-specific lineage (e.g. hg A2, B2, D1, C1b, C1c, C1d) was detected in Iceland.

As for Mesolithic Europe, the possibility of a direct prehistoric genetic influence from the Americas is highly unlikely. However, in the eventuality that further sampling of complete mtDNA genomes in the Americas reveals the presence of additional haplotypes belonging to C1f, it would suggest an evolutionary history similar to that of mtDNA hg X2. Like hg C1, hg X2 displays relatively low frequencies albeit with a global distribution in the Northern hemisphere. For example, clade X2a was observed in Europe in the West, in the Near East, Europe, Central Asia, Siberia as well as North America [43]. One model for the present-day distribution of hg X2 suggests that clade X2a split early from the rest of the X2 lineages in the Near East, and reached east Siberia before participating in the second wave of migration into the Americas through admixture with Beringian populations [44]. A similar scenario involving an early split of the different C1 clades in Asia followed by their spread and subsequently isolated evolution could be considered as an explanation for the wide geographical distribution of hg C1 in general. However, this scenario currently lacks substantial support.

### Similar Genetic Pre-history for the Icelandic-specific C1e and the Mesolithic C1f European Sub-clades

While the updated phylogeography of hg C1 does not allow defining the precise origins and divergence times of the C1f and C1e clades, the observation of C1f in Mesolithic Yuzhnyy Oleni Ostrov brings us to reconsider the hypotheses concerning the origins of C1e. Building on a hypothesis proposed by [10], we suggest that the Icelandic-specific C1e sub-clade could have had a recent origin in northern Europe rather than an American origin. This hypothesis is relevant with regard to the origins of the Icelandic population, as Iceland was discovered and first settled by Scandinavian Vikings around 1,130 years ago. Vikings raids extended as far from their homeland in Scandinavia as France, Spain and Sicily, but their main expansion range comprised western Russia, the Baltic region, Scandinavia, and the British Isles [16]. The study of the mtDNA diversity of present-day Icelanders identified that most of the Icelandic mtDNA lineages had Norse (from Scandinavia) or Gaelic origins (from the British Isles) and that the Icelandic gene pool had strongly been impacted by genetic drift [16]–[17], [45].

Considering the Scandinavian origins of Icelanders and the identification of the sister clade C1f in Mesolithic North East Europe, it can be proposed that the Icelandic-specific C1e and C1f sub-clades might have both split from the common ancestors of the C1 lineages somewhere in Eurasia and later reached northern Europe during independent or similar migrations (before the Mesolithic for C1f). Therefore, the rare occurrence of the C1e and C1f sub-clades in Europe could be the result of their dilution within the pre-existing European mtDNA diversity when these lineages reached Europe. Of note, a contrasting pattern of elevated frequency and diversity was observed for the American C1 sister-clades (C1b, C1c and C1d): all three American sub-clades signal important population expansion during the initial peopling of the continent, which was void of human occupation and thus competing lineages. The distribution of the C1e sub-clade restricted to Iceland, associated with the presence of the novel sub-clade C1f in a region neighbouring the homeland of Vikings and clearly predating the Viking expansion, lends support to the hypothesis that hg C1e might have been brought in by the Vikings who first colonised Iceland. The presence of a novel sub-clade (C1f) closely related to the Icelandic-specific C1e sub-clade in a region neighbouring the homeland of Vikings and clearly predating the Viking expansion lends support to the hypothesis that hg C1e might have been brought in by the Vikings who first colonised Iceland. While the C1e sub-clade might have been preserved at detectable frequencies in the Icelandic population due to the effects of founder event, it most likely has gone extinct in the source population in northern Europe as a consequence of its low frequency. In contrast, due to the small size of the population through time, Icelandic mtDNA diversity has been greatly affected by genetic drift and increased rates of mtDNA haplotype extinctions [45]. As such, the C1e clade would be more likely to survive in the potential North European source population than in Iceland [45], but the extensive sampling of the Icelandic population makes it more likely to be detected there than anywhere else in North Europe. The potential long-term survival of C1 lineages in prehistoric Europe is highly relevant to the discussion about the prehistoric interactions between the ancestral populations of Europeans, Siberians and Native Americans. It is consistent with recently published genomic data from a 24,000 year-old Upper Paleolithic individual from Mal’ta, South Siberia [46]. Interestingly, this individual was shown to belong to the western Eurasian hg U, which was also the most frequent hg found in Yuzhnyy Oleni Ostrov Mesolithic individuals (64%) [20]. Genome-wide data from Upper Palaeolithic Mal’ta revealed affinities with both present-day western Eurasian and Native Americans, and further supports gene-flow between the ancestral populations of Europeans and Native Americans prior to the colonisation of the Americas [46]. The new C1f lineage thus bridges the geographic gap between the Icelandic, the Siberian and the Native American C1 lineages and argues for the presence of C1 lineages, albeit at low frequency, in prehistoric West Eurasia.

## Materials and Methods

### Ethics Statement

No specific permits were required for the described field studies.

### Archaeological Samples

The three tooth samples analysed in this study were collected at the Mesolithic site of Yuzhnyy Oleni Ostrov, Onega Lake, Karelia, Russian Federation (61°30′N 35°45′E). These samples are under the custody of V.K. at the Peter the Great Museum of Anthropology and Ethnography (Kunstkamera) RAS, St Petersburg, Russian Federation, and were previously subjected to aDNA analyses in [20]. The three samples are identified as follows: UZOO-7 (MAE RAS collection number 5773–7, grave number 56), UZOO-8 (MAE RAS collection number 5773–8, grave number 57), and UZOO-74 (MAE RAS collection number 5773–74, grave number 114). Individuals UZOO-7 and UZOO-8 were found in adjacent graves (grave number 56 and grave number 57), whereas UZOO-74 was found in a grave located at the other end of the graveyard (grave number 114; see map in [47]).

### Ancient DNA Extraction

Among the three Mesolithic individuals from Yuzhnyy Oleni Ostrov shown to carry hg C1 (UZOO-7, UZOO-8, UZOO-74) in [20], individual UZOO-74 was selected for mtDNA genome sequencing on the basis of its subjectively good preservation and robust performance in previous Polymerase Chain Reaction (PCR) amplification experiments [20]. DNA extractions followed established protocols as described previously [20].

### Enrichment of Ancient Human Mitochondrial DNA

Ancient DNA extracts from specimens preserved in soil are expected to contain DNA molecules of various origins. In addition to the highly degraded DNA of the specimen under study, environmental, microbial DNA (bacteria and fungi), as well as from unidentified sources, has been shown to constitute a major proportion in the pool of DNA molecules present in aDNA extracts (e.g., [48]). The presence of a mixed population of DNA molecules from various organisms hampers the reliable sequencing of the DNA fragments of interest. Here, targeted enrichment of ancient human mtDNA of the extract for individual UZOO-74 was a crucial step prior to sequencing on the Ion Torrent PGM. Our aim was to increase the concentration of mtDNA fragments above the concentration threshold required for obtaining unambiguous sequencing at sufficient mtDNA genome coverage on an Ion Torrent PGM 316 chip. Ancient DNA libraries were enriched for human mtDNA using a hybridisation-based method described in [28]. All enrichment steps were performed twice for sample UZOO-74 to produce a ‘second round enrichment’ DNA library.

### Sequencing on the Ion Torrent PGM

The enriched library DNA was prepared for Ion Torrent sequencing by re-amplification using Ion Torrent barcoded adapters (Adapter A: 5′-CCATCTCATCCCTGCGTGTCTCCGACTCAGAAAAAGGTGTTGTTAGGAATGCGAGA-3′; Adapter B: 5′-CCTCTCTATGGGCAGTCGGTGATAGGATAGGTCGTTGCTGTGTA-3′). Eight reactions with a total volume of 25 µL were re-amplified using 1 µL of purified library DNA as template. Final reaction conditions comprised of 1x AmpliTaq Gold buffer II, 2.5 mM MgCl_2_, 2.5 U AmpliTaq Gold (Applied Biosystems), 250 µM of each dNTP (Invitrogen), and 0.5 µM of each PCR primer. The thermocycling profile consisted of 94°C for 12 min, followed by 12 cycles of 30 s at 95°C, 30 s at 60°C and 45 s at 72°C, followed by a final 10 min at 72°C. The eight PCRs were pooled and purified using MiniElute spin columns (Qiagen), then eluted into 15 µL as per the manufacturer’s instructions. The DNA was sized and quantified via gel electrophoresis against size markers (HyperLadder V, Bioline) and a Nanodrop 2000 (Thermo Scientific). Library DNA was size-selected above 120 bp and further purified, to remove adaptor dimers, using Qiagen’s gel extraction purification kit following manufacturer’s instructions.

Prior to sequencing, individual libraries were assessed for fragment size distribution and DNA concentration using a Bioanalyzer 2100 (Agilent Technologies) following manufacturer’s instructions. The quantified indexed library DNA was pooled to an equimolar concentration alongside other samples. The pooled library DNA was adjusted to a final concentration of 10–15 pM prior to amplification (by emulsion PCR) and enriched for positive Ion Sphere Particles (ISPs) using the Ion Torrent One Touch System II (Life Technologies) and the Ion One Touch 200 template kit v2 DL (Life Technologies), following manufacturer’s instructions. Templated ISPs were sequenced on a 316 micro-chip (up to 100 Mb of data) using the Ion Torrent Personal Genome Machine (PGM; Life Technologies) and the Ion PGM 200 sequencing kit v2 chemistry (Life Technologies) for 130 cycles (520 flows). After sequencing, the individual sequence reads were filtered within the PGM software to remove low quality and polyclonal sequences. Sequences matching the PGM 3′ adaptor were also automatically trimmed prior to bioinformatics analysis.

### Bioinformatics and Sequence Analysis

Next generation sequencing data (Ion Torrent PGM platform) from the mtDNA capture was processed using an in-house customizable analytical pipeline based on available scripts. Reads were de-multiplexed by barcode, not allowing for any mismatch, using FASTX-Toolkit (http://hannonlab.cshl.edu/fastx_toolkit/index.html). Cutadapt v1.1 [49] was then used to trim adapters and filter the reads for quality and length (five successive rounds of trimming with 33.3% error rate allowed; minimum quality value of Phred 20; minimum and maximum length of 25 and 110 bp after trimming). The filtered reads were checked with FastQC (http://www.bioinformatics.bbsrc.ac.uk/projects/fastqc), before mapping against the RSRS [8] using TMAP v3.2.1 (https://github.com/nh13/TMAP) with the following options: -g 3 -M 3 -n 7 -v stage1–stage-keep-all map1–seed-length 12–seed-max-diff 4 stage2 map2–z-best 5 map3–max-seed-hits 10. Mapped reads with mapping quality below Phred 30 and all duplicates were removed using both Samtools v0.1.18 [50] and the MarkDuplicates option of Picard Tools v1.79 (http://picard.sourceforge.net). GC content of mapped reads was analysed using the CollectGcBiasMetrics tool of Picard Tools v1.79. Misincorporation patterns were assessed using MapDamage v2.0.1 [10]–[11] and default parameters. The resulting sequence assembly was analyzed using Geneious Pro (v5.6.2) software in order to build the consensus sequence and assign a haplotype following the latest nomenclature and phylogeny on PhyloTree.org (Build 15 (30 Sep 2012)).

Genomic coverage by unique reads was compared to the distribution of GC content across the mtDNA using a custom R script (see Figure S1). Coverage depth was directly estimated from the unique read mapping file by counting the number of reads covering each mtDNA position. We also calculated the average GC percentage in a sliding window of 55 nt centered around each mtDNA position, taking in account the circular nature of the molecule. The size of the window corresponds to the average size of the unique mapped reads (see the Results section and Figure S2).

### SNP Confirmation by Direct Sequencing and Minisequencing

Selected regions of the mtDNA genome were sequenced independently via single and multiplex PCR amplification followed by direct sequencing (Table S1), and SNaPshot minisequencing following established PCR conditions [30] in order to:

1. verify the HVR-I sequence between np 15997 and 16410;
2. verify the 22 coding region hg diagnostic SNPs targeted by the GenoCoRe22 reaction;
3. interrogate the deletions at np 249, 290, 291, which are diagnostic of the CZ and C1 sub-haplogroups (Figure 3);
4. confirm the new SNPs identified here in the mtDNA genome sequence of specimen UZOO-74, but also to type these in the two other C1 carriers from Yuzhnyy Oleni Ostrov, UZOO-7 and UZOO-8.

Typing of coding-region SNPs using the GenoCore22 reaction was performed using the protocol described in [20].

### Authentication of the Ancient mtDNA Sequence

The aDNA sequence data was validated using three lines of evidence: 1) monitoring of contamination, 2) reproducibility, 3) phylogenetic consistency.

1. Pre-PCR DNA work was carried out at the Australian Centre for Ancient DNA (ACAD), University of Adelaide, a purpose-built positive air pressure laboratory dedicated to aDNA studies, which is physically isolated from any molecular biology laboratory amplifying DNA. Routine decontamination of the laboratory surfaces and instruments involves exposure to UV radiation and thorough cleaning using DNA oxidants such as bleach, Decon (Decon labs) and Isopropanol. In order to protect the laboratory environment from human DNA, researchers wear protective clothes including body suit, a facemask, a face shield, gumboots, and three layers of surgical gloves that are changed on a regular basis. Obvious large-scale contamination within the laboratory or in the reagents were monitored and controlled by blank controls (one extraction blank for every five ancient samples and two PCR/GenoCoRe22 blank controls for every six reactions). In addition, no haplotype similar to any of those possessed by laboratory members was consistently amplified from aDNA extracts.
2. For all three Yuzhnyy Oleni Ostrov individuals, the HVR-I sequences and GenoCoRe22 profiles could be replicated from two samples extracted independently [20]. For UZOO-74, the same HVR-I sequence and coding region SNPs were obtained by direct sequencing/GenoCoRe22 minisequencing and sequencing the complete mtDNA genome. Private SNPs identified by complete mtDNA genome sequencing were also confirmed by direct sequencing.
3. The phylogenetic consistency of the combination of variable positions in the mtDNA genome was an additional indicator of the authenticity of the sequence. The fifty-one nucleotide differences with RSRS were 100% consistent with the phylogenetic position of UZOO-74 on the hg C1 branch. No deviation from this position could be detected. The combination of five additional mutations was found to be unique to the haplotype sequenced here. None of these additional mutations was found to define branches within the human mtDNA tree, thus providing little support for them arising from contamination. In addition, considering the multiple replications performed to type the SNPs of interest, jumping PCR is thought to have had little or no impact on the hg C1 haplotype presented here.

### Phylogenetic Analysis of Haplogroup C1

In order to construct a phylogenetic network of the hg C1 HVR-I sequences displaying the C1 mutational pattern A16129G, T16187C, C16189T, G16230A, T16278C, T16298C, C16311T, T16325C, C16327T in Eurasia were gathered from the literature (Table S2). Sequences of whole mtDNA genomes belonging to hg C1 were also compiled from the online GenBank database on the basis of the list published in [10] (Table S2) and relevant new publications since. Sequences were corrected for known sequencing errors and ambiguities and, in particular, sequence length polymorphisms following recommendations in [39] and [51]. Mutations 309.1C, 315.1C, A16182c, A16183c, 16193.1C and C16519T were systematically ignored, as these positions are known to represent mutational hotspots and/or recurrent sequencing artefacts, which create reticulations in the phylogenetic analysis [35]. A tree was then constructed manually for complete mtDNA genome sequences on the basis of the tree constructed in [10] taking the latest mtDNA phylogeny into account (www.phylotree.org; [35]).

### Divergence Time Estimate

The split of the six clades from the hg C1 root could theoretically be dated using mtDNA genome sequences. However, the use of molecular data for dating divergence times can be problematic. The most commonly used method in modern mtDNA studies is based on the calculation of the ρ statistic [52]. However, this method has been shown to produce inaccurate dates, especially when the genetic data was collected from populations with complex demographic histories (e.g., [53]). The tree topology of hg C1 suggests that this is likely to be the case for the six C1 clades. The tree obtained is indeed very imbalanced: the three American clades show strong signals of a recent expansion, contrary to the three clades in Asia, Iceland and Mesolithic Yuzhnyy Oleni Ostrov. Moreover, a limitation of molecular dating methods is the inaccuracy of the estimation of the human mtDNA substitution rate and the fact that they rely on the use of a constant mutation rate through time (e.g., [54]). A recent study could largely improve the accuracy of such substitution rate estimates by correcting for purifying selection but still rely on a fossil calibration point, i.e., the human/chimp split ∼6.5 million years ago [55]. Based on this method, divergence time estimate were calculated for the modern sub-clades C1a, C1b and C1c at around 17,100 yBP (95% Confidence Interval: 12,000–22,500 yBP). The use of dated ancient mtDNA genome sequences to calibrate the molecular clock has so far not been considered by this method. However, recent studies have utilised ancient mtDNA genomes as tip calibrations in a Bayesian Markov Chain Monte Carlo framework using the program BEAST [56], which allows estimation of a substitution rate that is allowed to vary in time and can also take temporally heterogeneous mtDNA genome sequences into account [27]–[28]. However, the case of the polytomous hg C1 tree revealed problematic [7] it was recently demonstrated that a biased or incomplete representation of C1d lineages could lead to a much younger divergence time estimate (7,000–9,000 yBP; [7]). The addition of new data resulted in a revised divergence time to 18,700+/−1,400 yBP, in accordance with estimates for all other Pan-American haplogroups (15,000–18,000 yBP; [4], [7], [42], [57]). With only one C1f and very few Icelandic C1e mtDNA genomes, it is apparent that the sampling of the genetic diversity for these clades is not yet extensive enough to reliably calculate a divergence time estimates. Similarly, it seems also unreasonable to reconstruct the timing of the arrival of hg C1 lineages in Europe via coalescence age dating and/or determine whether all European C1 lineages reached Europe as part of the same migration as the Yuzhnyy Oleni Ostrov C1f branch or as part of other movements from the East.

### Accession Numbers

The deep-sequencing data were deposited on the NCBI Sequence Read Archive, accession number: SRP033724.

## Supporting Information

Figure S1 **Coverage depth and GC content for the haplogroup C1f mitochondrial genome (individual UZOO-74).** Mapping coverage of unique reads (in blue) is given per base. Local GC content (red) is shown for 55-bp intervals. The arrow indicate missing data between nucleotide positions 7277 and 7556, a region also characterized by low GC content.
(PDF)

Figure S2 **Analysis of DNA damage patterns.** A. Four upper plots: Frequencies of the A, C, G and T bases according to the nucleotide positions within the read (within the grey box) and outside the read (outside the grey box). Two lower plots: Frequency distribution of specific substitutions from the 5′-end (left) to the 3′-end (right) of the read sequence. B. Two upper plots: Read length distribution. Two lower plots: Observed cumulative frequency of C to T and G to A misincorporations. C. Observed frequencies of nucleotide misincorporation and simulated Bayesian posterior predictive intervals obtain from model fitting. D Simulated posterior distribution of model parameters: Lambda, probability of terminating in overhang (λ); DeltaD, probability of cytosine deamination in double strands (∂D); and DeltaS, probability of cytosine deamination in single strands (∂S).
(PDF)

Table S1 **Primer sequences.**
(PDF)

Table S2 **Haplogroup C1 mitochondrial sequences used to construct control region and complete mitochondrial genome C1 phylogenies.**
(PDF)
