# Supplementary figures and images for: Mitochondrial Genome Sequencing in Mesolithic North East Europe Unearths a New Sub-Clade within the Broadly Distributed Human Haplogroup C1
Source: PLoS One. 2014 Feb 4;9(2):e87612. doi: 10.1371/journal.pone.0087612 (PMC3913659; doi:10.1371/journal.pone.0087612)

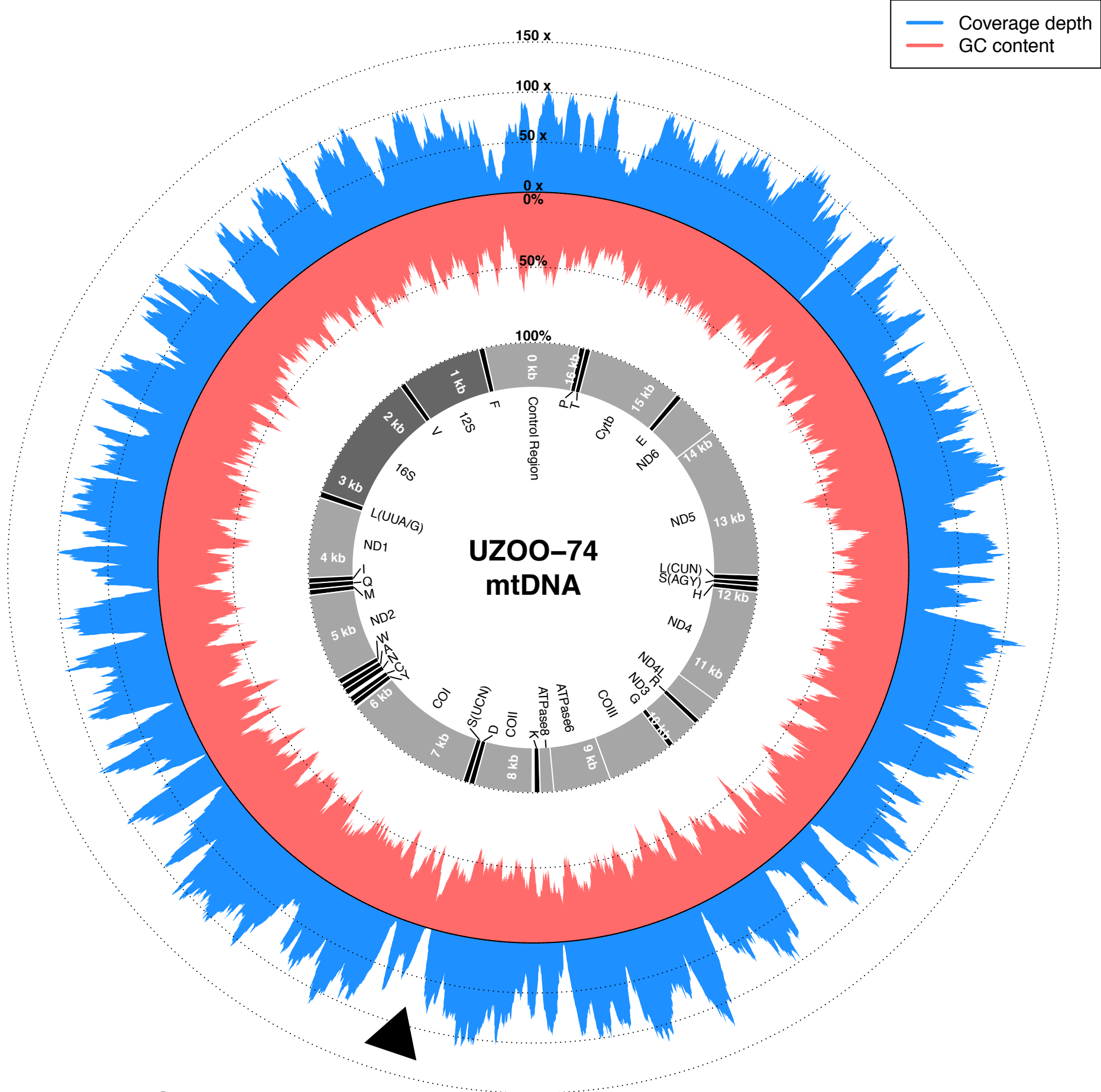

**Figure S1**

Supplement: Figure S1 — Coverage depth and GC content for the haplogroup C1f mitochondrial genome (individual UZOO-74). Mapping coverage of unique reads (in blue) is given per base. Local GC content (red) is shown for 55-bp intervals. The arrow indicate missing data between nucleotide positions 7277 and 7556, a region also characterized by low GC content. (PDF) [file pone.0087612.s001.pdf]

**A**

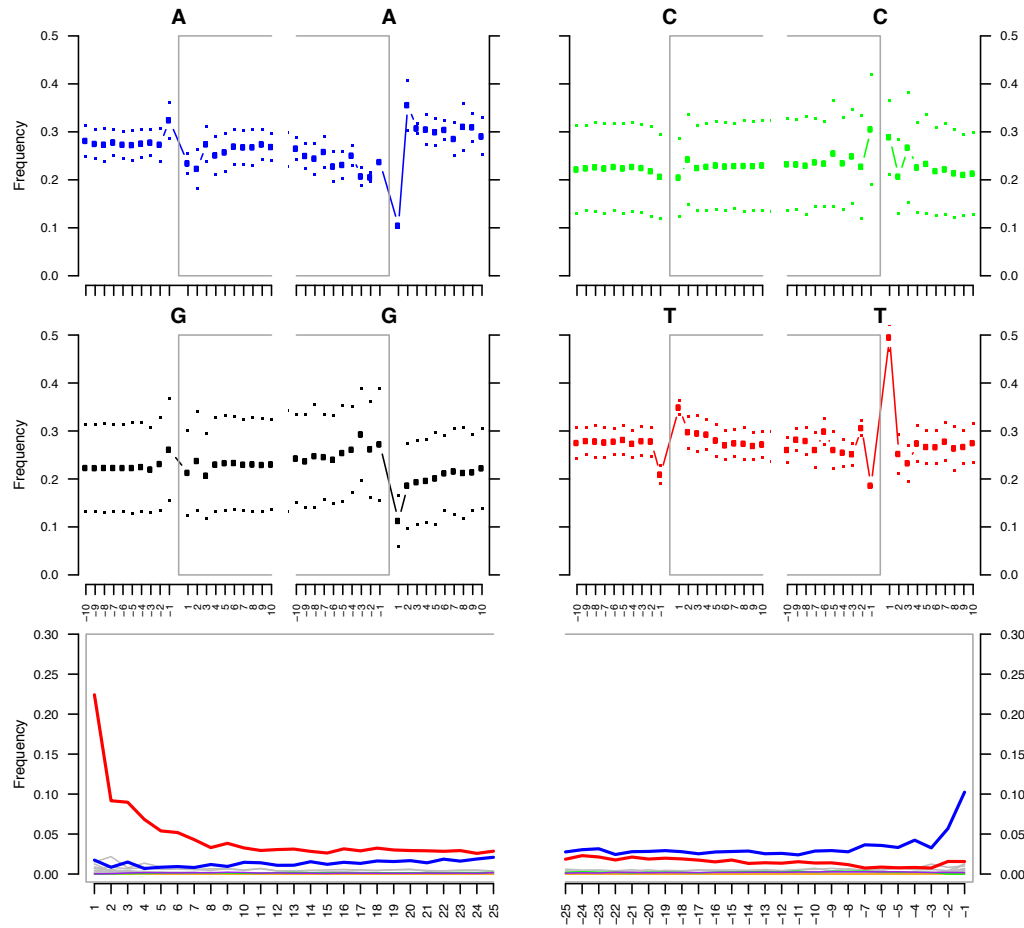

**B**

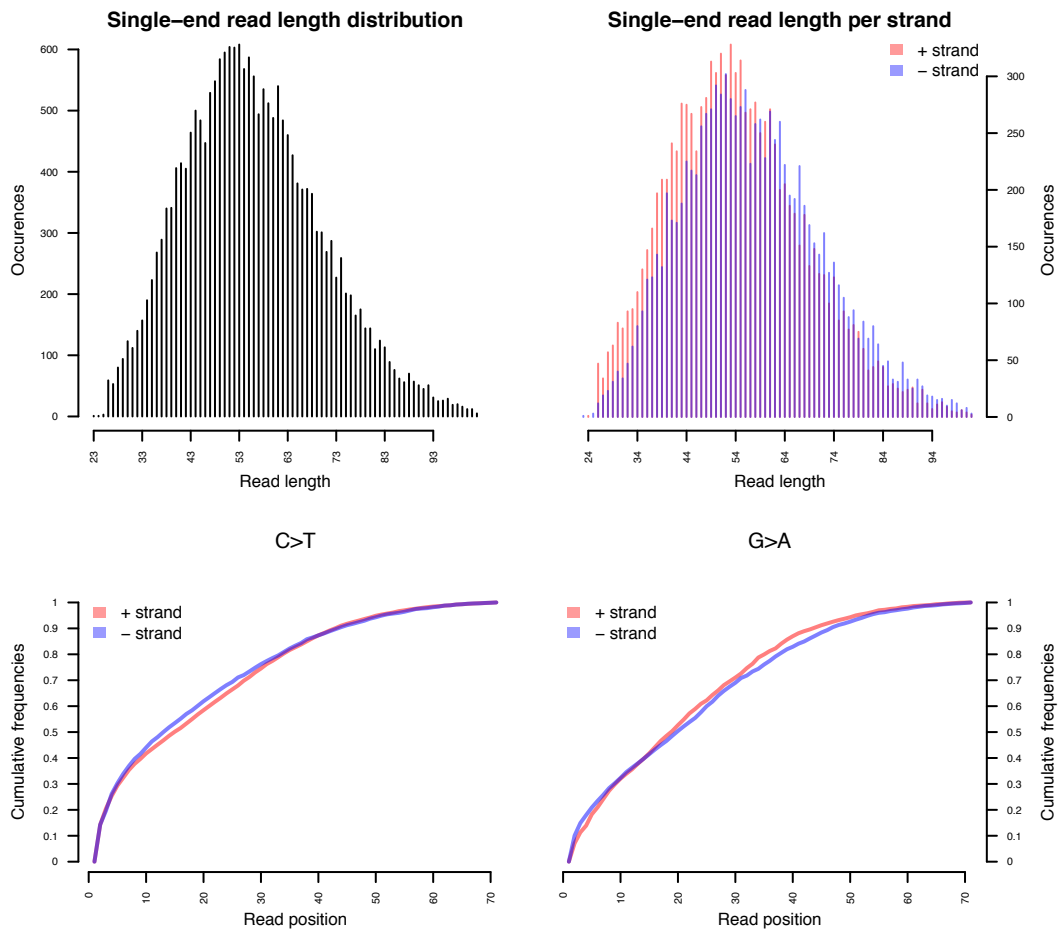

**C**

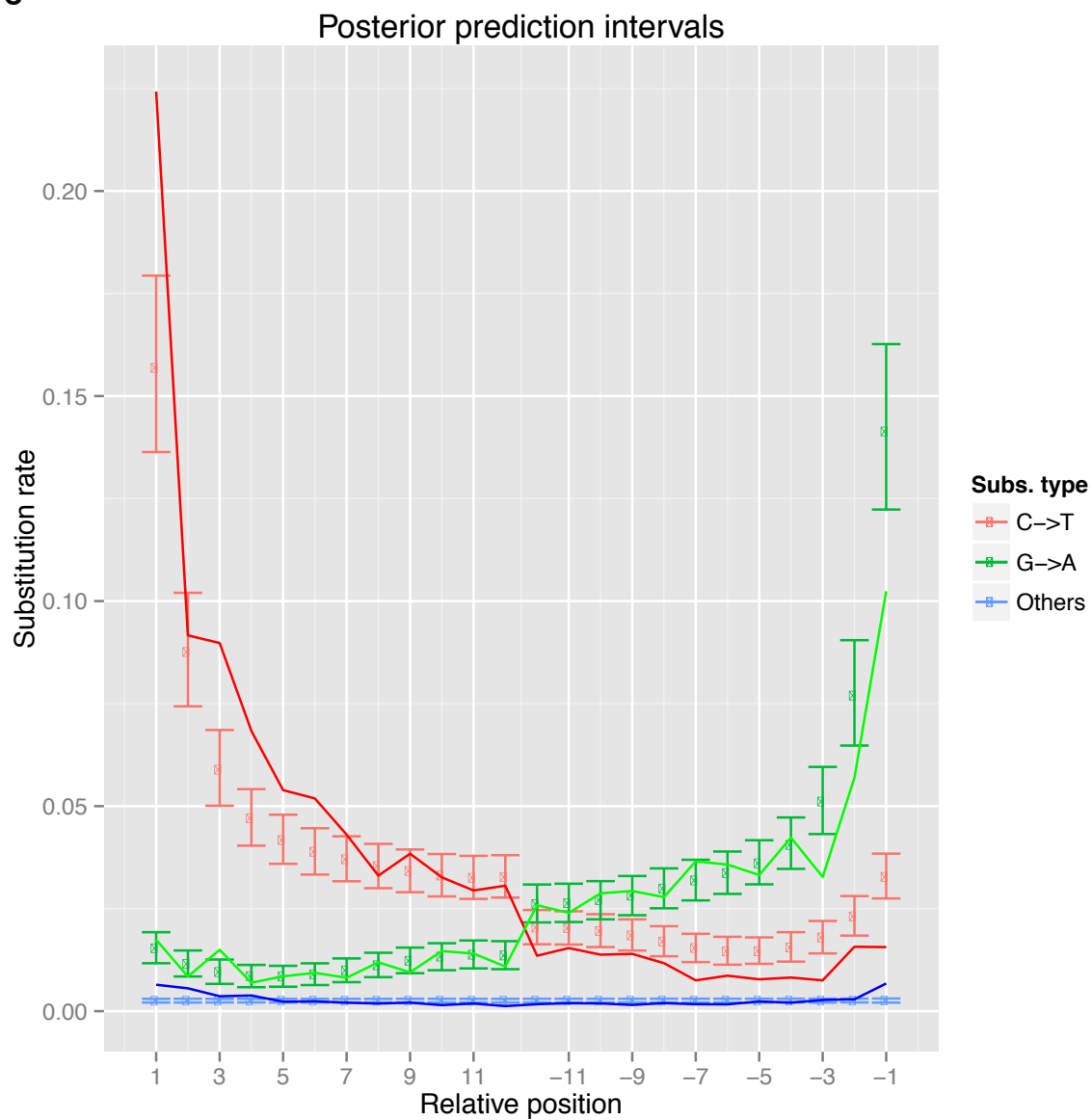

**D**

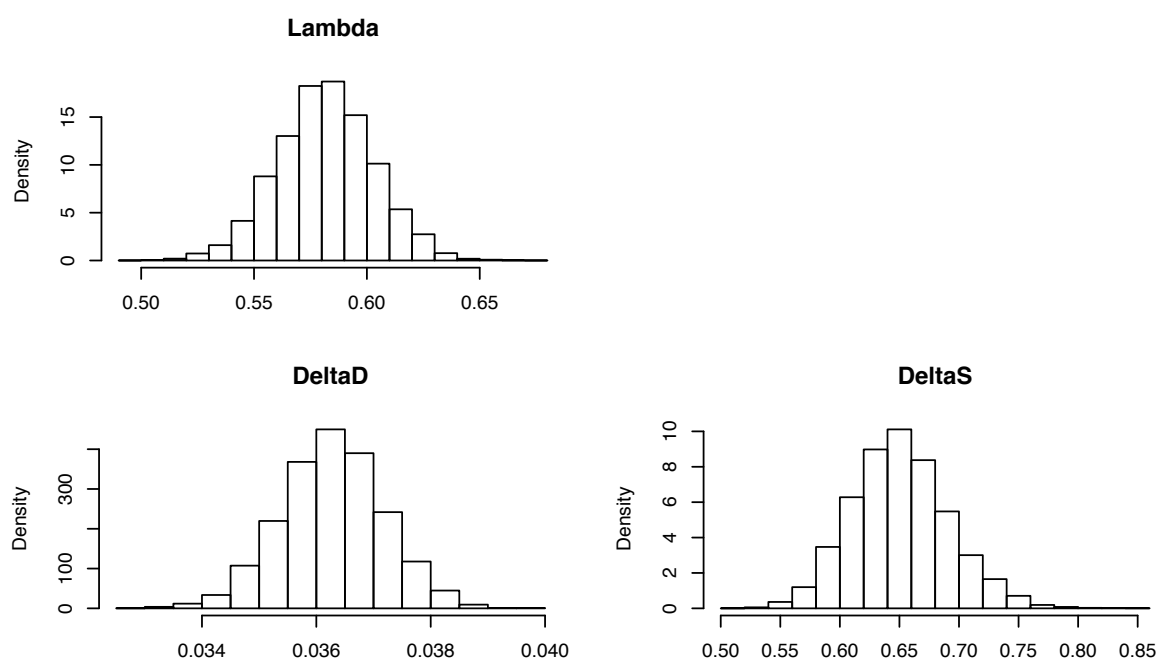

**Figure S2**

Supplement: Figure S2 — Analysis of DNA damage patterns. A. Four upper plots: Frequencies of the A, C, G and T bases according to the nucleotide positions within the read (within the grey box) and outside the read (outside the grey box). Two lower plots: Frequency distribution of specific substitutions from the 5′-end (left) to the 3′-end (right) of the read sequence. B. Two upper plots: Read length distribution. Two lower plots: Observed cumulative frequency of C to T and G to A misincorporations. C. Observed frequencies of nucleotide misincorporation and simulated Bayesian posterior predictive intervals obtain from model fitting. D Simulated posterior distribution of model parameters: Lambda, probability of terminating in overhang (λ); DeltaD, probability of cytosine deamination in double strands (∂D); and DeltaS, probability of cytosine deamination in single strands (∂S). (PDF) [file pone.0087612.s002.pdf]
