# Supplementary material for: Mitochondrial Genome Sequencing in Mesolithic North East Europe Unearths a New Sub-Clade within the Broadly Distributed Human Haplogroup C1
Source: PLoS One. 2014 Feb 4;9(2):e87612. doi: 10.1371/journal.pone.0087612 (PMC3913659; doi:10.1371/journal.pone.0087612)
Supplement: Table S1 — Primer sequences. (PDF) [file pone.0087612.s003.pdf]

Table S1. Primer sequences.

			Primer name	Primer sequence (5' - 3')	Positions of the targeted fragment	Fragment size (base pair)	Reference
**Hypervariable Region I**							
			L15996	CTCCACCATTAGCACCCAAAGC	15977 - 16161	185	[1]
			H16142	ATGTACTACAGGTGGTCAAG			[2]
			L16117	TACATTACTGCCAGCCACCAT	16097 - 16237	141	[3]
			H16218	TGTGTGATAGTTGAGGGTTG			[4]
			L16209	CCCCATGCTTACAAGCAAGT	16190 - 16322	133	[4]
			H16303	TGGCTTTATGTACTATGTAC			[4]
			L16287	CACTAGGATACCAACAAACC	16268 - 16429	162	[4]
			H16410	GCGGGATATTGATTTCACGG			[4]
**Hypervariable Region II**							
			L00205	ACAGGCGAACATACTTACTAAAGTG	00181 - 00261	105	This study
			H00261	GTTATGATGTCTGTGTGGAAAGTGG			This study
			L00270	TGAATGTCTGCACAGCCACTTTCCA	00246 - 00350	105	This study
			H00327	TTGGCAGAGATGTGTTTAAGTGCT			[5]
**Coding Region**							
	**Haplogrou**	**Targeted SN**	**Primer name**	**Primer sequence (5' - 3')**	**Positions of the targeted fragment**	**Fragment size (base pair)**	**Reference**
	C1f	8577	L8562	TTCGCTTCATTCATTGCCCCC	08542 - 08624	110	This study
			H8624	AGTCGGTTGTTGATGAGATATTTGGAGG			This study
	C	9545	L9524	CTGAGCCTTTTACCACTCCAGCCTA	09500 - 09559	83	This study
			H9559	GATTTAGCGGGGTGATGCCTGTTG			This study
	C1f	11605	L11595	ACAAGCTCCATCTGCCTACGACA	11573 - 11631	80	This study
			H11631	ACTACGAGGGCTATGTGGCTGA			This study
	C1f	12217	L12197	TGACAACAGAGGCTTACGACCCC	12175 - 12197	68	This study
			H12223	TGGGGGCATGAGTTAGCAGT			This study

REFERENCES

1. Vigilant L, Stoneking M, Harpending H, Hawkes K, Wilson AC. (1991) African populations and the evolution of human mitochondrial DNA. Science 253(5027):1503-7.
2. Stone AC, Stoneking M. (1998) mtDNA analysis of a prehistoric Oneota population: implications for the peopling of the New World. Am J Hum Genet 62(5):1153-70.
3. Haak W, Forster P, Bramanti B, Matsumura S, Brandt G, et al. (2005) Ancient DNA from the first European farmers in 7500-year-old Neolithic sites. Science 310: 1016-1018.
4. Handt O, Krings M, Ward RH, Pääbo S. (1996) The retrieval of ancient human DNA sequences. Am J Hum Genet 59(2):368-76.
5. Haak W, Brandt G, de Jong HN, Meyer C, Ganslmeier R, et al. (2008) Ancient DNA, Strontium isotopes, and osteological analyses shed light on social and kinship organization of the Later Stone Age. Proc Natl Acad Sci U S A 105(47):18226-31.
