# Supplementary material for: Mitochondrial Genome Sequencing in Mesolithic North East Europe Unearths a New Sub-Clade within the Broadly Distributed Human Haplogroup C1
Source: PLoS One. 2014 Feb 4;9(2):e87612. doi: 10.1371/journal.pone.0087612 (PMC3913659; doi:10.1371/journal.pone.0087612)
Supplement: Table S2 — Haplogroup C1 mitochondrial sequences used to construct control region and complete mitochondrial genome C1 phylogenies. (PDF) [file pone.0087612.s004.pdf]

Table S2. Haplogroup C1 mitochondrial sequences used to construct control region and complete mitochondrial genome C1 phylogenies.

C1 clade	Geographic /Ethnic origin	Reference	GenBank accession number
**Hypervariable Region I sequences**			
C1?	Lebanese	[1]	
C1?	Lebanese	[1]	
C1?	Bashkir	[2]	
C1?	Bashkir	[2]	
C1?	Bashkir	[2]	
C1?	Icelander	[3]	
C1?	Icelander	[3]	
C1?	Icelander	[4]	
C1?	Thai	[5]	
C1?	Indian	[6]	
C1?	Indian	[6]	
C1?	Indian	[6]	
C1?	Indian	[6]	
C1?	Indian	[6]	
C1?	Indian	[6]	
C1?	Germany	[7]	
C1?	Canary Islander	[8]	
C1a	Altaian	[1]	
C1a	Altaian	[1]	
C1a	Orok	[9]	
C1a	Orok	[9]	
C1a	Orok	[9]	
C1a	Orok	[9]	
C1a	Orok	[9]	
C1a	Orok	[9]	
C1a	Orok	[9]	
C1a	Highland Kirghiz	[10]	
C1a	Japanese	[11]	
C1a	Japanese	[12]	
C1a	Japanese	[12]	
C1a	Mongolian	[13]	
C1a	Mongolian	[13]	
C1a	Daur	[14]	
C1a	Korea	[15]	
C1a	Kalmyk	[16]	
C1a	Kalmyk	[16]	
C1a	Buryat	[17]	
C1a	Buryat	[17]	
C1a	Buryat	[17]	
C1a	Buryat	[17]	
C1a	Buryat	[17]	

C1 clade	Geographic / Ethnic origin	Reference	GenBank accession number
C1a	Buryat	[17]	
C1a	Ulchi	[18]	
C1a	Starikovskaya	[18]	
C1a	Japanese	[19]	
C1a	Buryat	[19]	
C1a	Japanese	[20]	
**Complete mitochondrial genomes**			
C1a	Japanese	[20]	AP008311
C1a	Ulchi, Siberia	[18]	AY519496
C1a	Buryat, Siberia	[21]	EF153779
C1a	Nanaitci, Siberia	[22]	EU007858
C1b	Canary	[23]	AF382009
C1b	North America	[24]	AY195759
C1b	Hispanic	[25]	DQ282447
C1b	Hispanic	[25]	DQ282448
C1b	Hispanic	[25]	DQ282449
C1b	Hispanic	[25]	DQ282450
C1b	Hispanic	[25]	DQ282451
C1b	Hispanic	[25]	DQ282452
C1b	Hispanic	[25]	DQ282453
C1b	Hispanic	[25]	DQ282454
C1b	Hispanic	[25]	DQ282455
C1b	Hispanic	[25]	DQ282456
C1b	Hispanic	[25]	DQ282457
C1b	Hispanic	[25]	DQ282458
C1b	Hispanic	[25]	DQ282461
C1b	Hispanic	[25]	DQ282464
C1b	Hispanic	[25]	DQ282469
C1b	Hispanic	[25]	DQ282475
C1b	Hispanic	[25]	DQ282476
C1b	South America, Zoro	[26]	EU095223
C1b	South America, Zoro	[26]	EU095224
C1b	South America, Quechua,	[26]	EU095225
C1b	South America, Quechua	[26]	EU095226
C1b	Brazil, Arara	[26]	EU095227
C1b	South America, Poturujara,	[26]	EU095228
C1b	Brazil/Venezuela, Yanomamö	[26]	EU095229
C1b	Brazil/Venezuela, Yanomamö	[26]	EU095230
C1b	Brazil/Venezuela, Yanomamö,	[26]	EU095231
C1b	Colombia/Venezuela, Wayuu	[27]	EU095549
C1b	United States	[28]	EU431085
C1b	Mexico, Pima	[29]	EU597545

C1 clade	Geographic / Ethnic origin	Reference	GenBank accession number
C1b	Mexico, Pima	[29]	EU597557
C1b	Mexico	[30]	HQ012197
C1b	Mexico	[30]	HQ012236
C1b	Mexico	[30]	HQ012210
C1b	Mexico	[30]	HQ012202
C1b	Mexico	[30]	HQ012208
C1b	Mexico	[30]	HQ012212
C1b	Unknown	[31]	JQ702984
C1b	Unknown	[31]	JQ705451
C1b	Unknown	[31]	JQ701891
C1b	Unknown	[31]	JQ705153
C1b	Chile	[32]	JX413036
C1b	Chile	[32]	JX413037
C1b	Chile	[32]	JX413038
C1b	Chile	[32]	JX413039
C1b	Chile	[32]	JX413040
C1b	Chile	[32]	JX413041
C1b	Chile	[32]	JX413042
C1b	Chile	[32]	JX413043
C1b	Chile	[32]	JX413044
C1b	Chile	[32]	JX413045
C1b	Chile	[32]	JX413046
C1b	Chile	[32]	JX413047
C1b	Chile	[32]	JX413048
C1b	Chile	[32]	JX413049
C1b	Chile	[32]	JX413050
C1b	Chile	[32]	JX413051
C1b	Chile	[32]	JX413052
C1b	Chile	[32]	JX413053
C1b	Chile	[32]	JX413054
C1b	Chile	[32]	JX413055
C1b	Chile	[32]	JX413056
C1c	South America, Arsario	[27]	EU095527
C1c	Colombia, Kogui	[27]	EU095544
C1c	Dominican Republic	[28]	EF079875
C1c	Canada	[28]	EU431086
C1c	United States	[28]	EU431087
C1c	Hispanic	[25]	DQ282459
C1c	Hispanic	[25]	DQ282460
C1c	Hispanic	[25]	DQ282462
C1c	Hispanic	[25]	DQ282463
C1c	Hispanic	[25]	DQ282465

C1 clade	Geographic / Ethnic origin	Reference	GenBank accession number
C1c	Hispanic	[25]	DQ282466
C1c	Hispanic	[25]	DQ282467
C1c	Hispanic	[25]	DQ282468
C1c	Hispanic	[25]	DQ282470
C1c	Hispanic	[25]	DQ282471
C1c	Unknown	[33]	EU327891
C1c	Unknown	[33]	EU327973
C1c	Unknown	[33]	EU617323
C1c	Mexico	[30]	HQ012230
C1c	Mexico	[30]	HQ012223
C1c	Unknown	[31]	JQ704040
C1c	Unknown	[31]	JQ705574
C1c	Unknown	[31]	JQ703827
C1d	Venezuela/Guyana, Warao	[34]	AF347012
C1d	Venezuela/Guyana, Warao	[34]	AF347013
C1d	Colombia, Corequaje	[27]	EU095537
C1d	Hispanic	[25]	DQ282472
C1d	Hispanic	[25]	DQ282473
C1d	Hispanic	[25]	DQ282474
C1d	Brazil/Guyana, WaiWai	[26]	EU095222
C1d	Canada, British Columbia	[35]	GU215075
C1d	Colombia, Mestizos	[36]	HM107313
C1d	Colombia, Mestizos	[36]	HM107314
C1d	Colombia, Mestizos	[36]	HM107315
C1d	USA, Montana	[36]	HM107319
C1d	Mexico, Zacatecas	[36]	HM107321
C1d	Mexico, Sonora	[36]	HM107322
C1d	Argentina, Salta	[36]	HM107323
C1d	Argentina, Buenos Aires,	[36]	HM107327
C1d	Argentina, Buenos Aires	[36]	HM107329
C1d	Mexico, Oaxaca	[36]	HM107334
C1d	USA, Texas,	[36]	HM107335
C1d	Argentina, Buenos Aires	[36]	HM107346
C1d	Uruguay	[36]	HM107348
C1d	Chile, Bio-bío	[36]	HM107350
C1e	Icelandic	[37]	
C1e	Icelandic	[37]	
C1e	Icelandic	[37]	

References

1. Balanovsky, personnal coommunication

2. Bermisheva M, Tambets K, Villems R, Khusnutdinova E (2002) Diversity of mitochondrial DNA haplotypes in ethnic populations of the Volga-Ural region of Russia. Mol Biol 36: 990-1001.

3. Helgason A, Sigureðardóttir S, Gulcher JR, Ward R, Stefánsson K (2000) mtDNA and the origin of the Icelanders: deciphering signals of recent population history. Am J Hum Genet 66:999-1016.

4. Helgason A, Nicholson G, Stefánsson K, Donnelly P (2003) A reassessment of genetic diversity in Icelanders: strong evidence from multiple loci for relative homogeneity caused by genetic drift. Ann Hum Genet 67:281-297.

5. Kutanan W, Kampuansi J, Fuselli S, Nakbunlung S, Seieslstad M et al. (2011) Genetic structure of the Mon-Khmer speaking groups and their affinity to the neighbouring Tai populations in Northern Thailand. BMC Genet. 12:56.

6. Metspalu M, Kivisild T, Metspalu E, Parik J, Hudjashov G, et al. (2004) Most of the extant mtDNA boundaries in south and southwest Asia were likely shaped during the initial settlement of Eurasia by anatomically modern humans. BMC Genet 5:26.

7. Pfeiffer H, Brinkmann B, Hühne J, Rolf B, Morris A A, et al. (1999) Expanding the forensic German mitochondrial DNA control region database: genetic diversity as a function of sample size and microgeography. Int J Legal Med 112:291-298.

8. Rando JC, Cabrera VM, Larruga JM, Hernandez M, Gonzalez AM, et al. (1999). Phylogeographic patterns of mtDNA reflecting the colonization of the Canary Islands. Ann Hum Genet 63:413-428.

9. Bermisheva MA, Kutuev IA, Spitsyn VA, Villems R, Batyrova AZ, et al. (2005) Analysis of mitochondrial DNA variation in the population of Oroks. Genetika 41:78-84.

10. Comas D, Calafell F, Mateu E, Pérez-Lezaun A, Bosch E, et al. (1998) Trading genes along the silk road: mtDNA sequences and the origin of Central Asian populations. Am J Hum Genet 63:1824-1838.

11. Horai S, Hayasaka K (1990) Intraspecific nucleotide sequence differences in the major noncoding region of human mitochondrial DNA. Am J Hum Genet 46:828-42.

12. Imaizumi K, Parsons TJ, Yoshino M, Holland MM (2002) A new database of mitochondrial DNA hypervariable regions I and II sequences from 162 Japanese individuals. Int J Legal Med 116:68-73.

13. Kolman CJ, Sambuughin N, Bermingham E (1996) Mitochondrial DNA analysis of Mongolian populations and implications for the origin of New World founders. Genetics 142:1321-34.

14. Kong Q P, Yao Y G, Sun C, Bandelt H J, Zhu C L, Zhang Y P (2003) Phylogeny of East Asian mitochondrial DNA lineages inferred from complete sequences. Am J Hum Genet 73: 671-676.

15. Lee HY, Yoo JE, Park MJ, Chung U, Shin KJ (2006) Mitochondrial DNA control region sequences in Koreans: identification of useful variable sites and phylogenetic analysis for mtDNA data quality control. Int J Legal Med 120:5-14.

16. Nasidze I, Quinque D, Dupanloup I, Cordaux R, Kokshunova L, et al. (2005) Genetic evidence for the Mongolian ancestry of Kalmyks. Am J Phys Anthropol 128:846-54.

17. Shimada MK, Kim C, Takahashi A, Saitou N, Suzuki M. (2002) Mitochondrial DNA control region sequences for a Buryats population in Russia. DNA Polymorphism 10:150-155.

18. Starikovskaya EB, Sukernik RI, Derbeneva OA, Volodko NV, Ruiz-Pesini E, et al. (2005) Mitochondrial DNA diversity in indigenous populations of the southern extent of Siberia, and the origins of Native American haplogroups. Ann Hum Genet 69:67-89.
19. Tajima A, Hayami M, Tokunaga K, Juji T, Matsuo M, et al. (2004) Genetic origins of the Ainu inferred from combined DNA analyses of maternal and paternal lineages. J Hum Genet 49:187-93.
20. Tanaka M, Cabrera VM, González AM, Larruga JM, Takeyasu T, et al. (2004) Mitochondrial genome variation in eastern Asia and the peopling of Japan. Genome Res 14:1832-50.
21. Derenko M, Malyarchuk B, Grzybowski T, Denisova G, Dambueva I, et al. (2007) Phylogeographic analysis of mitochondrial DNA in northern Asian populations. Am J Hum Genet 81: 1025-41.
22. Ingman M, Gyllensten U (2007) A recent genetic link between Sami and the Volga-Ural region of Russia. Eur J Hum Genet 15: 115-120.
23. Maca-Meyer N, González AM, Larruga JM, Flores C, Cabrera VM (2001) Major genomic mitochondrial lineages delineate early human expansions. BMC Genet 2:13.
24. Mishmar D, Ruiz-Pesini E, Golik P, Macaulay V, Clark AG, et al. (2003) Natural selection shaped regional mtDNA variation in humans. Proc Natl Acad Sci U S A 100: 171-176.
25. Just RS, Diegoli TM, Saunier JL, Irwin JA, Parsons TJ. (2008) Complete mitochondrial genome sequences for 265 African American and U.S. "Hispanic" individuals. Forensic Sci Int Genet 2(3):e45-8.
26. Fagundes NJ, Kanitz R, Bonatto SL. (2008) A reevaluation of the Native American mtDNA genome diversity and its bearing on the models of early colonization of Beringia. PLoS One 3(9):e3157
27. Tamm E, Kivisild T, Reidla M, Metspalu M, Smith DG, et al. (2007) Beringian standstill and spread of Native American founders. PLoS One 2(9):e829.
28. Achilli A, Perego UA, Bravi CM, Coble MD, Kong QP, et al. (2008) The phylogeny of the four pan-American MtDNA haplogroups: implications for evolutionary and disease studies. PLoS One 3(3):e1764.
29. Hartmann A, Thieme M, Nanduri LK, Stempfl T, Moehle C, et al. (2009) Validation of

microarray-based resequencing of 93 worldwide mitochondrial genomes. Hum Mutat. 30(1):115-122.

30. Kumar S, Bellis C, Zlojutro M, Melton PE, Blangero J, et al. Large scale mitochondrial sequencing in Mexican Americans suggests a reappraisal of Native American origins. BMC Evol Biol. 11:293.

31. Behar DM, van Oven M, Rosset S, Metspalu M, Loogväli EL, et al. (2012) A "Copernican" reassessment of the human mitochondrial DNA tree from its root. Am J Hum Genet 90(4):675-84.

32. de Saint Pierre M, Gandini F, Perego UA, Bodner M, Gómez-Carballa A, et al. (2012) Arrival of Paleo-Indians to the southern cone of South America: new clues from mitogenomes. PLoS One 7(12):e51311.

33. Family Tree DNA database.

34. Ingman M, Kaessmann H, Pääbo S, Gyllensten U (2000) Mitochondrial genome variation and the origin of modern humans. Nature 408: 708-713.

35. Malhi RS, Cybulski JS, Tito RY, Johnson J, Harry H, et al. (2010) Brief communication: mitochondrial haplotype C4c confirmed as a founding genome in the Americas. Am J Phys Anthropol. 141(3):494-497.

36. Perego UA, Angerhofer N, Pala M, Olivieri A, Lancioni H, et al. (2010) The initial peopling of the Americas: a growing number of founding mitochondrial genomes from Beringia. Genome Res. 20(9):1174-1179.

37. Ebenesersdóttir SS, Sigurðsson A, Sánchez-Quinto F, Lalueza-Fox C, Stefánsson K, et al. (2011) A new subclade of mtDNA haplogroup C1 found in Icelanders: evidence of pre-Columbian contact? Am J Phys Anthropol. 144(1):92-99.
